# The Importance of the Pyrazole Scaffold in the Design of Protein Kinases Inhibitors as Targeted Anticancer Therapies

**DOI:** 10.3390/molecules28145359

**Published:** 2023-07-12

**Authors:** George Mihai Nitulescu, Gheorghe Stancov, Oana Cristina Seremet, Georgiana Nitulescu, Dragos Paul Mihai, Cosmina Gabriela Duta-Bratu, Stefania Felicia Barbuceanu, Octavian Tudorel Olaru

**Affiliations:** Faculty of Pharmacy, Carol Davila University of Medicine and Pharmacy, Traian Vuia 6, 020956 Bucharest, Romania; george.nitulescu@umfcd.ro (G.M.N.);

**Keywords:** adenine-mimetic, ATP-competitive, asciminib, avapritinib, crizotinib, encorafenib, erdafitinib, pralsetinib, pirtobrutinib, ruxolitinib

## Abstract

The altered activation or overexpression of protein kinases (PKs) is a major subject of research in oncology and their inhibition using small molecules, protein kinases inhibitors (PKI) is the best available option for the cure of cancer. The pyrazole ring is extensively employed in the field of medicinal chemistry and drug development strategies, playing a vital role as a fundamental framework in the structure of various PKIs. This scaffold holds major importance and is considered a privileged structure based on its synthetic accessibility, drug-like properties, and its versatile bioisosteric replacement function. It has proven to play a key role in many PKI, such as the inhibitors of Akt, Aurora kinases, MAPK, B-raf, JAK, Bcr-Abl, c-Met, PDGFR, FGFRT, and RET. Of the 74 small molecule PKI approved by the US FDA, 8 contain a pyrazole ring: Avapritinib, Asciminib, Crizotinib, Encorafenib, Erdafitinib, Pralsetinib, Pirtobrutinib, and Ruxolitinib. The focus of this review is on the importance of the unfused pyrazole ring within the clinically tested PKI and on the additional required elements of their chemical structures. Related important pyrazole fused scaffolds like indazole, pyrrolo[1,2-b]pyrazole, pyrazolo[4,3-b]pyridine, pyrazolo[1,5-a]pyrimidine, or pyrazolo[3,4-d]pyrimidine are beyond the subject of this work.

## 1. Introduction

The protein kinases (PKs) represent a large family of enzymes that catalyze the transfer of a phosphate group, released by adenosine triphosphate (ATP), to the hydroxyl group of serine, threonine or tyrosine residues of their protein substrates [1]. Protein phosphorylation is an essential cellular mechanism of regulation with high impact on signal transduction, cell growth and proliferation [2]. In general, PKs can function on multiple substrates and various proteins can be phosphorylated by more than one specific kinase [3]. The aberrant activation (or overexpression) of PKs has been frequently observed in cancer cells and represents a major mechanism of tumoral development, making PKs the focus of extensive research and the most widely-studied therapeutic targets in the field of oncology [4]. Protein kinase inhibitors (PKIs) have emerged as valuable therapeutic options in the treatment of various cancers, and their development has radically transformed the field of targeted cancer therapy [5].

PKIs encompass a diverse range of chemical structures, as different compounds have been developed to interact selectively with specific enzymes but share some common structural features or scaffolds that are essential for the target binding and inhibitory activity [6,7,8]. The concept of privileged scaffolds was introduced to describe chemical frameworks that have shown broad activity against multiple targets within a specific target family [9,10]. The scaffolds are not limited to a single target family, but they have demonstrated a higher propensity for successful drug discovery within that particular target class [11]. In the context of PKs, there are several privileged scaffolds that have proven effective in developing kinase inhibitors, such as the pyrazolo[3,4-d]pyrimidine scaffold [12,13,14], the imidazo[1,2-b]pyridazine scaffold [15] or the indazole ring [16]. Several structural modifications of these heterocycles are needed to enhance the binding affinity and selectivity of the inhibitors [1].

The pyrazole scaffold is widely used in medicinal chemistry and drug discovery, and it represents an important building block in the development of PKIs, being a key privileged scaffold [17,18,19]. The literature provides several in-depth reviews focused on the design, synthesis, and biological evaluation of pyrazole-based anticancer agents, highlighting the pharmacological potential of this scaffold [20,21,22,23], but takes little notice of its propensity toward the family of PKs.

The objective of this study was to present, in a critical manner, the importance of the unfused pyrazole ring in the structures of clinically tested PKIs, and to accentuate the additional structural requirements. The notion “unfused pyrazole” was used to avoid the confusion with fused pyrazoles, because many authors incorrectly use the term “pyrazole” for both types of compounds. The study tries to offer chemists the proper pharmacological background in order to select the appropriate therapeutic target or biological pathway they wish to investigate for their synthesized pyrazoles.

The review is structured based on the primary target of the pyrazole-based inhibitors detailing the specifics of each class. Table S1 presents a summary of the clinical assays performed on the reviewed compounds and their approval status with the Food and Drug Administration (FDA) and the European Medicines Agency (EMA). The completed/suspended studies were not included in the table.

## 2. The Chemical Profile of the Pyrazole Ring

Pyrazole is a compound from the five-membered heterocycles class, with two nitrogen heteroatoms in vicinal positions. This diazole heterocycle has aromatic character, presenting a system of six π electrons, four of them coming from three carbon atoms and one nitrogen atom, hybridized sp^2^, with each contributing one electron from the unhybridized p orbital [24]. The second nitrogen atom provides a pair of electrons located in a p orbital that is coplanar with the other p orbitals. The extended molecular orbital is formed by the interpenetration of these atomic orbitals stabilizing the heterocyclic system [25,26]. Due to its pronounced aromatic character, pyrazole can participate in electrophilic substitution reactions (nitration, sulfonation, halogenation) in position 4. Positions 3 and 5 are deactivated because of the presence of electronegative nitrogen atoms, facilitating nucleophilic attacks at these last two positions [27].

The substitutions on the pyrazole ring have a major impact on their chemical and biological properties. The N-unsubstituted pyrazoles derivatives present amphoteric properties, due to the pyrrole-like nitrogen (NH) that can easily donate its proton, while the pyridine-like nitrogen atom (N) is capable of accepting protons. In general, the basic character is prevalent, but the presence of electron donating groups on the ring can increase the acidity of the NH group [26,28].

In terms of medicinal chemistry, the N-unsubstituted pyrazole ring is capable of simultaneously donating and accepting hydrogen bonds, while the substitution at the pyrrole-like nitrogen abolishes the acidic character and the capacity of the heterocycle to serve as a hydrogen bond donor [28,29]. In contrast to the related heterocyclic structures imidazole, thiazole, and oxazole, which tend to undergo metabolic oxidative cleavage to electrophilic fragments, the drugs containing pyrazole rings exhibit higher stability against oxygenases, such as P450. This stability is likely attributed to the strong acidic nature of pyrazole, which renders its derivatives less susceptible to oxidative metabolism. Drugs incorporating N-substituted pyrazoles as part of their structure frequently experience the removal of the substituent attached to the pyrazole ring [30].

## 3. Protein Kinases Structure and Inhibition Mechanisms

Both receptor protein kinases and non-receptor protein kinases share structural features specific to the PK superfamily. The main difference between the two subclasses is represented by the presence of a transmembrane segment and an extracellular segment (needed for ligand-dependent activation) in the case of receptor tyrosine kinases. The structure of PKs can be divided 12 subdomains (I-VIa, VIb-XI) or into N-terminal and C-terminal lobes, which are connected by a hinge region [31,32].

The N-terminal lobe is smaller and consists of five-stranded antiparallel β-sheet, an αC-helix occurring in both active and inactive conformations, and a glycine-rich loop (GRL or P-loop) that connects the β1- and β2-strands [33]. One exception to this generalization is represented by proto-oncogene kinase Pim-1, which has an extra beta-hairpin in the N-terminal lobe [34]. A common feature regarding the primary structure of PKs is the presence of a valine residue situated after the GRL, which is involved in hydrophobic interactions with both the adenine fragment of ATP and various scaffolds specific to small molecule PKIs [33]. The conserved β5-strand contains a bulky “gatekeeper” residue, which is located adjacent to the hinge region and distal to the active site. This residue regulates the interaction of PKs and nucleotides or small molecule PKI [35]. Mutations at this site can lead to treatment resistance by preventing the binding of some competitive PKIs, while other molecules can bypass this issue, acting as mutation resistant PKIs (e.g., T338M mutant c-Src kinase is resistant to type I inhibitor dasatinib, but not type II inhibitor RL45) [36]. Interestingly, mutations of the gatekeeper residue in ERK2 MAP kinase enhance autophosphorylation, elevating the kinase activity, and promote the binding of PP1 derivatives and N_6_-cyclopentyl ATP [35,37,38].

PKs can be found in either αC_in_ (active) or αC_out_ (inactive) conformations. The αC_in_ architecture is required for catalytic activity and is defined by the presence of a salt-bridge between a positively charged lysine within the β3-strand and a negatively charged glutamate within the αC-helix. Therefore, enzymatic activity cannot occur without the conversion of the αC_out_ conformation to the αC_in_ architecture [33,39].

The secondary structure of the C-terminal lobe is characterized by eight conserved α-helices (αD- αI, αEF1, αEF2) and four short β-strands (β6–β9). Interestingly, the second amino acid residue of the β7 strand is involved in hydrophobic interactions with all small molecules ATP-competitive PKI and is considered the “floor” of the adenine binding pocket. Moreover, the C-terminal lobe is involved in the positioning of the protein substrate into the active site and has a catalytic loop that mediates the γ-phosphoryl group transfer from ATP to substrates [33,40,41].

PKs have a highly conserved catalytic loop, which contains an HRD (His/Arg/Asp) motif. Enzymatic activity is dependent on a catalytic tetrad (K/E/D/D—Lys/Glu/Asp/Asp). The lysine of the tetrad is the same residue that forms a salt bridge with the αC-glutamate but is also involved in salt bridge formation with α- and β-phosphates of ATP [33]. The aspartate of the HRD sequence is the same as the first aspartate in the catalytic tetrad and functions as a proton acceptor (Lowry-Brønsted base), abstracting the proton from the substrate -OH [42]. The protein-substrate-binding activation segment starts with the second aspartate of the catalytic tetrad and is followed by a phenylalanine and a glycine (DFG pattern). The catalytic activity of many protein kinases is dependent on two Mg^2+^ ions, which are bound by the DFG aspartate and the terminal asparagine from the catalytic loop [33,43].

The activation segment is highly variable among the protein kinase superfamily and contains phosphorylatable residues, with their phosphorylation being mandatory for enzymatic activity (with few exceptions). Moreover, the activation segment has an open conformation in functional kinases and a closed conformation in inactive kinases. In active conformations, the DFG aspartate points toward the nucleotide binding site and binds the Mg^2+^ cation (DFG_in_), while in inactive conformations, the aspartate residue points away from the nucleotide binding site (DFG_out_). In DFG_in_ conformation, the phenylalanine sidechain is packed against or under the αC-helix, while in DFG_out_ conformation the phenylalanine is oriented into the ATP binding site. In a third, intermediate conformation called DFG_inter_ (or DFG_up_), the phenylalanine divides the active site into two halves, the side chain being out of the αC-helix and pointing towards the β-sheets [33,39]. The DFG_up_ state was first reported in an Aurora A kinase triple-point mutant that mimics the active site of Aurora B kinase bound to an inhibitor [44].

Currently, PKIs are categorized into 7 classes (I–VII), according to their mechanisms of action and binding modes. Types I–V are reversible inhibitors, while types VI-VII m are irreversible binders. Type I inhibitors interact with the enzyme in the active DFG_in_ conformation at the ATP binding site, while type II inhibitors bind to the inactive DFG_out_ conformation. Both type I and II molecules are competitive inhibitors. A subclass of type I inhibitors, called type I½, bind to the enzyme in DFG_in_ and αC_out_ conformation. Type III and IV PKIs are allosteric inhibitors that bind either within the ATP pocket (type III) or the substrate-binding domain (type IV). Type V inhibitors are bivalent molecules that target both the ATP binding pocket and substrate-biding domains. Lastly, type VI PKIs are covalent inhibitors that react primarily with nucleophilic cysteines within the ATP binding pocket or other sites proximal to the kinase domain, while type VII PKIs are nonclassical allosteric inhibitors that bind covalently to the extracellular domain of receptor PKs [45,46,47].

## 4. Akt Inhibitors

Akt kinase, also known as protein kinase B, is a serine/threonine kinase that plays a crucial role in cell survival, growth, proliferation, metabolism, and protein synthesis. It is a key component of the phosphatidylinositol 3-kinase (PI3K) signaling pathway, which is frequently dysregulated in cancer [48]. It consist of three isoforms (Akt 1–3) that share a high degree of structural homology and similar functions but have some distinct roles in specific tissues [49,50].

**Figure 1:**
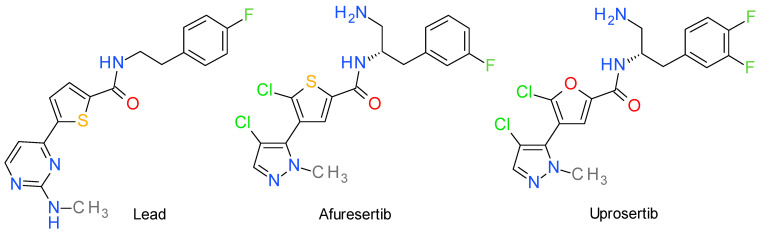
The structures of Akt inhibitors Afuresertib, Uprosertib and the lead structure of their development, the N-(2-phenylethyl)-5-pyrimidin-4-yl-thiophene-2-carboxamide derivate.

The most explored strategy was to target the ATP binding site, but despite its high efficiency to generate potent inhibitors, this approach makes the development of selective agents challenging because of the high homology of Akt with other PKs. The structures of representative pyrazole-based Akt inhibitors are presented in Figure 1.

### 4.1. Afuresertib

A high throughput screening identified a N-(2-phenylethyl)-5-pyrimidin-4-yl-thiophene-2-carboxamide derivative as an ATP-competitive inhibitor of Akt3. The structure activity relationships highlighted the importance of the amide bond and revealed the distance of two carbon atoms between the phenyl ring and the amide nitrogen as optimal [51]. Replacement of the 2-aminopyrimidine fragment with a pyrazole ring improved the effect and was the first step towards the development of afuresertib (GSK2110183) [52]. The pyrazole moiety is critical, providing only a single hydrogen bond with the hinge region of the kinase. The halogen-substituted benzene ring is also important by forming interactions with the hydrophobic pocket under the P-loop [53].

Afuresertib highly inhibits all three isoforms of Akt, with a higher potency against Akt1, with a cited half-maximal inhibitory concentration (IC_50_) value of 0.02 nM compared to Akt2 and Akt3 (IC_50_ values of 2 nM, respectivlly 2.6 nM). The mechanism is ATP-competitive and fully reversible [52]. Afuresertib is under evaluation in combination with other therapies in various clinical trials [54,55].

### 4.2. Uprosertib

Uprosertib (synonyms: GSK2141795, GSK795) is an ATP-competitive, orally bioavailable Akt inhibitor structurally similar to afuresertib, with the main difference being the replacement of the thiophene fragment with a furan ring [56]. Uprosertib is undergoing clinical investigation to treat triple-negative breast cancer [57].

## 5. Aurora Kinases Inhibitors

**Figure 2:**
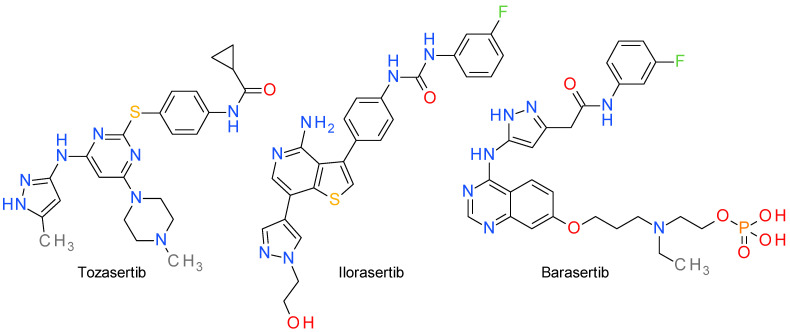
The structures of Aurora inhibitors Tozasertib, Ilorasertib, and Barasertib.

Aurora kinases are a group of three (AurA, AurB, AurC) serine/threonine protein kinases that play crucial roles in regulating various aspects of cell division, particularly in the process of mitosis. They are tightly regulated throughout the cell cycle to ensure accurate chromosome segregation and proper cell division [58]. AurA promotes the centrosome maturation and the mitotic spindle assembly preparing the transition from the G2 phase to the M phase of the cell cycle. AurB and AurC regulate the dynamic interactions between the chromosomes and the microtubules, ensuring proper chromosome alignment and segregation [59,60]. The inhibitors are usually classified as selective toward a specific Aurora isoform and nonselective (pan-Aur) inhibitors. The pyrazole template emerged as an important scaffold in the design of both non-selective inhibitors and subtype selective inhibitors [61]. The structures of clinically important pyrazole-based inhibitors are presented in Figure 2.

### 5.1. Tozasertib

Tozasertib, also known as VX-680 or MK-0457, is a pan-Aur inhibitor, with some degree of selectivity towards AurA. It has shown activity against a broad range of tumor types, including both solid tumors and hematological malignancies. It was evaluated in several clinical trials, the best response being observed in patients with leukemia [62].

An aminopyrazolyl substituted quinazoline derivative was identified as a lead in a screening campaign to find pan-Aur inhibitors. Tozasertib was developed by structural optimization of this lead compound mainly by using pyrimidine as a structural simplification core quinazoline scaffold [63]. The 3-aminopyrazole scaffold is a well-established adenine-mimetic pharmacophore, and in the case of tozasertib establishes hydrogen bonds with Glu211 and Ala213 in the hinge region. The binding of the aminopyrazole fragment is similar with that of the related pyrrolopyrazole derivative, danusertib. The carbonyl group of the cyclopropylamide fragment is also important for the interaction with the kinase [64,65].

Several structurally similar compounds were developed based on tozasertib model, such as ENMD-2076, an orally active potent inhibitor of AurA and of other cancer-related kinases [66,67], and AT9283, a 4-pyrazolamine derivative with broad-spectrum activity against a variety of kinases [68,69].

### 5.2. Ilorasertib

Ilorasertib, previously known as ABT-348, is a potent and ATP-competitive multitargeted kinase inhibitor with nanomolar potency against AurB and AurC, and micromolar potency against AurA. Ilorasertib inhibits, also, the vascular endothelial growth factor receptor (VEGFR) and the Src family of cytoplasmic tyrosine kinases [70]. Clinical evidence indicates that the inhibition of VEGFR2 is obtained at lower doses compared with the inhibition of Aurora kinsases [71].

The importance of the pyrazole ring in the structure of ilorasertib was revealed in the process of lead optimization. In the effort to increase the AurB inhibitory potency of a 4-amino-thienopyridine derivative, a series of aromatic rings were added in the position 7 of the heterocycle. Benzene, furan, and thiophene had little effect, while 3-pyrrole and 3-pyrazole had a medium impact. The best results were achieved with the pyrazole ring substituted in position 4. Docking studies confirmed the role of the pyrazole moiety that fits into the extended-hinge region of the enzyme [72].

### 5.3. Barasertib

Barasertib, also known as AZD1152, is a prodrug that is rapidly converted by the seric phosphatases to the active barasertib-hQPA, also referred to in the literature as defosbarasertib and AZD1152-HQPA, a potent and selective AurB inhibitor [73]. There are many articles that seem to confuse barasertib with its active form barasertib-hQPA or fail to clearly present it as such.

Barasertib is pyrazolyl-aminoquinazoline derivative for which the importance of the pyrazole scaffold was demonstrated in its lead optimization stage. The drug development process started with an anilino-quinazoline derivative that presented sub-micromolar potency against both AurA and AurB. The benzene ring was changed with several rings, such as pyrimidine, thiazole, and pyrazole. The pyrazole fragment was preferred because it afforded both potent inhibitors and also less lipophilic compounds with better drug-like properties [74].

Barasertib was evaluated in several clinical assays focused on various cancer types, such as acute myeloid leukemia, relapsed or refractory diffuse B-cell lymphoma, and small-cell lung cancer. The results indicated positive responses to the treatment and an acceptable toxicity profile [75,76].

## 6. MAPK Inhibitors

**Figure 3:**
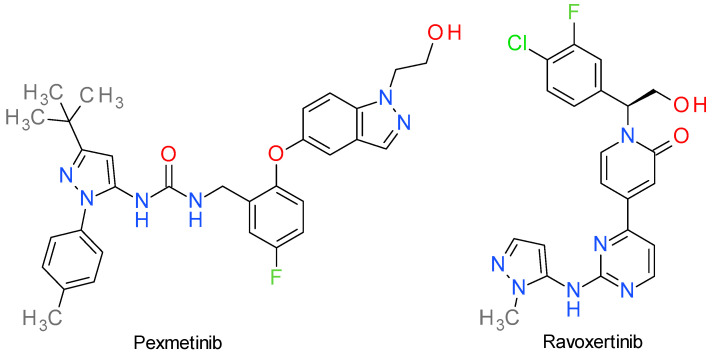
The structures of MAPK inhibitors Pexmetinib and Ravoxertinib.

Mitogen-activated protein kinases (MAPKs) are a family of PKs that play a critical role in cellular physiology, signaling, and various diseases. The three major subfamilies of MAPKs are the extracellular signal-regulated kinases (ERKs), p38 MAPKs, and c-Jun N-terminal kinases (JNKs). Dysregulation of the MAPKs signaling pathways is associated with several diseases, including cancer, cardiovascular disorders, neurodegenerative diseases, and inflammatory diseases [77]. The structures of two compounds are presented in Figure 3.

### 6.1. Pexmetinib

Pexmetinib, also referred in the literature as ARRY-614, is a potent, orally bioavailable, dual p38 MAPK and angiopoietin-1 receptor (Tie-2) inhibitor. There is little available recent literature that reviews its structure-analysis relationship, but it is known that the compound is a type II PKI that binds both p38 MAPK and Tie-2 kinase in the DFG_out_ conformation [78].

Chemically it is a pyrazoyl urea derivative and is very structurally similar to doramapimod (synonym: BIRB-796), a very potent allosteric inhibitor p38 MAPK [79]. Predominantly, doramapimod has an immunomodulator profile and has been studied for its potential therapeutic use in rheumatoid arthritis, chronic obstructive pulmonary disease, and other inflammatory disorders [80]. Based on the chemical similarities of these compounds, we consider that they share the same binding mechanism, with the *t*-butyl group on the pyrazole occupying the lipophilic domain exposed upon rearrangement of the activation loop (DFG_out_) [81]. Acumapimod (BCT197) is another pyrazole-based inhibitor of p38, but it is beyond the subject of this review because it is developed as an anti-inflammatory agent [82].

Pexmetinib inhibited both p38 MAPK and Tie-2 in nanomolar ranges in various cell-based systems and it was efficacious in preclinical tumor xenografts in mice models of chronic myelogenous leukemia and multiple myeloma at doses ranging from 30 to 100 mg/kg twice a day, orally [78]. It is currently under phase Ib and II clinical trials for possible treatment of renal cell cancer, melanoma, solid tumors, and myelodysplastic syndrome [83].

### 6.2. Ravoxertinib

Ravoxertinib (GDC-0994) is a potent, reversible, ATP-competitive, and highly selective ERK1 and ERK2 inhibitor, with IC_50_ of 6.1 nM and 3.1 nM, respectively. It inhibits ERK phosphorylation and activation of ERK-mediated signal transduction pathways, thus preventing ERK-dependent tumor cell proliferation and survival [84].

Ravoxertinib’s development began with a pyrimidinyl-pyridone derivative identified as a selective ERK1/2 inhibitor. This lead compound exhibited several desirable qualities, but also presented certain problematic characteristics. Notably, it had a very high human dose projection (>1 g/day), primarily due to metabolism occurring at the tetrahydropyran ring bound to the pyrimidine fragment. The replacement of the tetrahydropyran with heteroaromatic rings improved the metabolic stability and conserved the hydrogen bond with Lys114 of ERK2. The use of aminopyridine was detrimental because of the CYP3A4 inhibitory effect, but substituted 4-aminopyrazoles and 5-aminopyrazole provided good results. The N-methyl-5-aminopyrazole was chosen based on its higher cell potency and metabolic stability [85].

The X-ray crystallography data indicated that 2-aminopyrimidine binds to ERK2 by hydrogen bonds with Met108 and Leu107 in the hinge region, while the pyrazole ring bonds to Lys114. The methyl bound to the pyrazole ring is essential for the selectivity towards cyclin-dependent kinase 2 (CDK2) by sterically compromising the binding interactions. Other important binding elements are the pyridone carbonyl, the hydroxymethyl group, and the fluorochlorophenyl fragment [85].

In a phase I dose escalation study conducted in patients with locally advanced or metastatic solid tumors, ravoxertinib exhibited both an acceptable safety profile and desirable pharmacodynamic effects [86].

## 7. B-Raf Inhibitors

The RAF (Rapidly Accelerated Fibrosarcoma) kinases have been discovered more than two decades ago and comprise a family of three serine/threonine PKs, indexed with prefixes A to C [87]. The RAS-RAF-MEK-ERK cascade, crucial for various cellular processes such as regulation of cell cycle (including apoptosis), cell differentiation and proliferation [88], involving all the members of this kinase family, but, among them, B-Raf (expressed by the chromosome 7 located oncogene BRAF) [89] is the PK frequently altered in multiple cancer types, with notable mutation rates observed in skin, thyroid, gastrointestinal (GI), and lung cancers [90].

The best studied activating BRAF mutations occur at position V600 (V600E, V600K), the most common being the V600E mutation, characterized by the substitution of valine with glutamic acid at position 600 of the kinase domain. A BRAF mutation classification model breaks down three classes: class I (independent of upstream RAS signaling; activate the downstream ERK pathway without requiring dimerization), class II (require dimerization to activate the MEK-ERK pathway; independent of RAS signaling), and class III (downstream signaling through dimerization with wild-type CRAF; rely on upstream activation through genomic alterations or RTK signaling) [89,91]. Of the three classes, only compounds that target the first type of mutations have demonstrated clear clinical benefit.

The use of first-generation RAF inhibitors, such as dabrafenib or vemurafenib, led to better understanding the mechanisms of resistance and shed light on the importance of homo- or heterodimerization of B-Raf and C-Raf as critical in intrinsic or drug-induced resistance [92].

### Encorafenib

Encorafenib, sometimes identified as LGX818, is an EMA and FDA approved, highly potent RAF inhibitor. Just as with the multikinase inhibitor sorafenib, encorafenib competitively binds to the ATP socket domain of the kinases, with selective anti-proliferative and apoptotic activity in cells expressing B-Raf, especially those harboring the V600E mutation [91]. As opposed to dabrafenib, it presents the benefit of an increased half-life and increased potency against wild-type B-Raf and C-Raf (with a K_i_ of 0.3 nM). Structurally, amongst other small molecule inhibitors that target B-Raf, it presents a high hydrogen acceptor to donor ratio (10:3), with a high number of rotatable bonds (10) [93].

As with other PKIs, it has been postulated that for the effective inhibition of both the kinase domains of the protein, DFG_in_ and DFG_out_, B-Raf inhibitors should possess an appropriately sized linker between the hydrogen bond donor part of the molecule and the hydrogen bond accepter [94].

**Figure 4:**
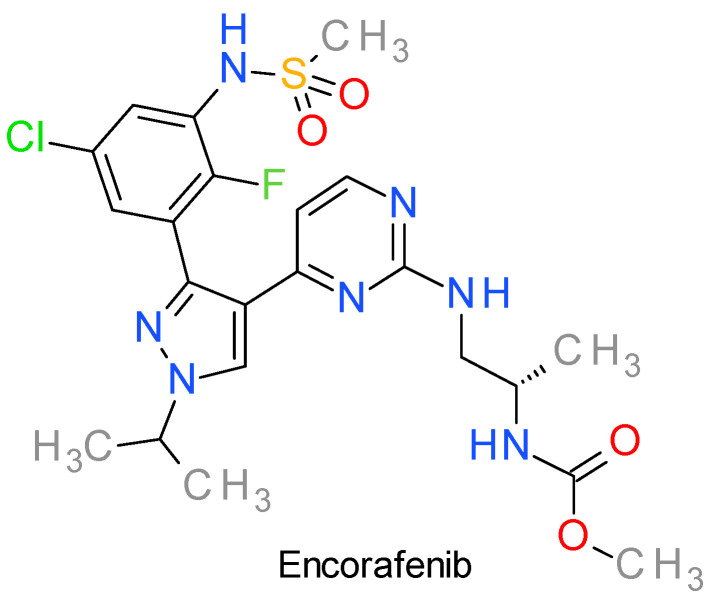
The structure of Encorafenib.

Diving forward into the structural analysis of small molecule B-raf inhibitors, 1,3,4-triarylpyrazole derivatives similar to encorafenib have been synthetized and evaluated as having a promising inhibitory and thus antiproliferative potential, especially those with a sulfonamide functional group [95,96,97]. Moreover, the two aryl rings at positions 3 and 4 of encorafenib’s pyrazole scaffold (Figure 4) also play an important role in binding to the active kinase domain, through hydrophobicity and hydrogen bonding valences [98]. However, in another study that investigated imidazothiazole derivatives as B-raf V600E inhibitors, the strategy of replacing the hydrophobic fluoro group with the more hydrophilic nitro group managed to secure both the electrophilic and the hydrogen bond acceptor characteristics of the aromatic substituent, thus increasing the kinase binding ability, possibly revealing insights on the presence and importance of the sulfonamide moiety (alongside the two halogen groups on the aromatic ring at position 3) in the design of encorafenib [99].

There are several clinical trials focused on evaluating encorafenib in various cancer types, the majority of them in combination with other anticancer agents (Table S1). The combination of encorafenib with binimetinib, a MEK inhibitor, was approved by FDA in 2018 for patients with advanced melanoma harboring the BRAF V600 mutation [100]. In 2020, the FDA granted approval for the utilization of encoratinib in combination with cetuximab to treat metastatic colorectal cancer characterized by the BRAF-V600E mutation, following prior treatment [101].

## 8. Inhibitors of Various Other Serine/Threonine Kinases

**Figure 5:**
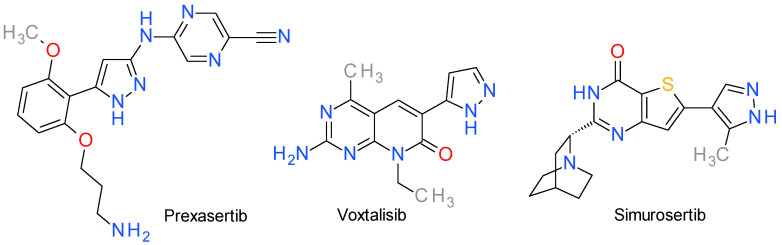
The structures of Prexasertib, Voxtalisib, and Simurosertib.

This section groups a series of pyrazole derivatives that inhibit various serine/threonine kinases. Their structures are presented in Figure 5.

### 8.1. Prexasertib

Prexasertib (investigational name: LY2606368) is an ATP-competitive, second-generation checkpoint kinase (CHK) 1 inhibitor that blocks DNA repair of cancer cells, leading to the accumulation of damaged DNA and, consequently, cell apoptosis [102]. The IC_50_ value on CHK1 is less than 1 nM, and the assay on a broad panel of other 224 PKs revealed that only CHK2 and the RSK family kinases presented an IC_50_ under 10 nM (8 nM and 9 nM, respectively) [103].

Structurally, it contains an aminopyrazine core and a 2,6 dialkoxy phenyl group linked by the pyrazole ring. A molecular docking study revealed that prexasertib develops four binding interactions with CHK1 (pdb 7AKM), a strong hydrogen donor bond with Glu91, two weak hydrogen acceptor bonds toward Lys38 and Lys132, and a hydrophobic interaction with Leu15 [104].

Prexasertib is currently being evaluated in phase I and II clinical trials both in mono-therapy and in combination with other drugs for the treatment of advanced or metastatic cancer, including ovarian, breast or prostate cancers, brain tumors, head and neck squamous cell carcinoma, and small cell lung cancer [102].

### 8.2. Voxtalisib

Voxtalisib, referred also as XL765, is a dual inhibitor of mTOR and phosphoinositide 3-kinase (PI3K). It was developed manner, with a weaker effect on mTOR2. An analysis of sensitivity based on genotypes through the refinement of a pyridopyrimidinone derivative to achieve effective inhibition of the PI3K/mTOR pathway in living organisms, while maintaining desirable drug-like characteristics. It inhibits various class I PI3K isoforms and mTOR1 in an ATP-competitive indicated that PIK3CA-mutant cell lines demonstrated a higher degree of sensitivity towards voxtalisib, whereas cell lines with RAS or BRAF mutations tended to exhibit lower sensitivity [105]. The literature concerning these compounds is sometimes confusing and should be carefully analyzed because the code XL765 is also used for a (3,5-dimethoxyphenyl)aminoquinoxaline derivative developed as a PI3K/mTOR inhibitor [106].

### 8.3. Simurosertib

Cell division cycle 7 (Cdc7) is a serine/threonine kinase that gained considerable interest as a promising target in cancer therapy because it plays a vital role in the initiation and preservation of DNA replication in eukaryotic cells [107].

Simurosertib, also known as TAK-931, is a targeted inhibitor of Cdc7 with a time-dependent and ATP-competitive mechanism. It has been chosen as an advanced, replication stress-inducing anticancer drug, due to its ability to extend replication stress and induce subsequent mitotic abnormalities. It inhibits the proliferation in both in vitro and in vivo preclinical cancer models, showcasing its unique activity spectrum, especially against cancer cell lines carrying RAS mutations [108].

Development of this compound started with a thieno[3,2-d]pyrimidin-4(3H)-one derivative, serendipitously identified as a Cdc7 inhibitor. The replacement of the pyridine fragment bound in position 6 with a 3-methylpyrazole ring significantly enhanced the potency. The presence of the methyl group on the pyrazole ring is notably significant, contributing not only to high potency but also to time-dependency and a slow dissociation [109]. Several modifications at position 2 on the thienopyridine scaffold improved the potency and lead to simurosertib [110].

According to the docking study, it binds to the Cdc7 kinase through several key interactions, such as the hydrogen bond between Lys90 and the carbonyl group and the hydrogen bonds between Pro135 and Lys137 with the nitrogen atoms in the pyrazole ring [110].

In the first clinical trial in patients with solid tumors, simurosertib demonstrated that it was overall well-tolerated and had a manageable safety profile. Based on these findings, the recommended dose for phase II trials was determined to be 50 mg, administered once daily, from days 1 to 14 of each 21-day treatment cycle [111].

## 9. JAK Inhibitors

Janus kinases (JAKs) are a family of four non-receptor tyrosine kinases: JAK1, JAK2, JAK3, and Tyk2 (tyrosine kinase 2). They play a critical role in transmitting signals from various cytokine and growth factor receptors to the nucleus, regulating gene expression and controlling cellular processes, such as cell growth, differentiation, and immune responses. The dysregulation of the JAK/STAT pathway is associated with various types of cancers and autoimmune diseases [112]. JAK1 has an important role in inflammatory diseases caused by aberrant autoimmune responses, while JAK2 is correlated with oncologic pathologies [113].

**Figure 6:**
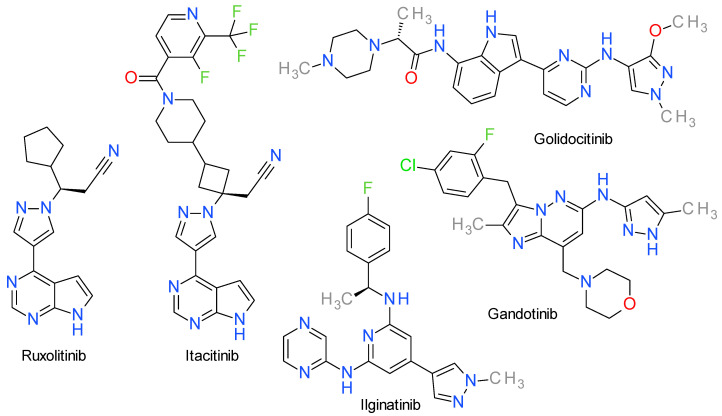
The structures of JAKs inhibitors Ruxolitinib, Itacitinib, Golidocitinib, Gandotinib, and Ilginatinib.

Several important mutations in JAKs, especially JAK2, are correlated with oncologic processes because they cause a disruption of the auto-inhibitory function, rendering JAKs constitutively active. These gain-of-function mutations are highly prevalent in myeloproliferative neoplasms, making JAKs a highly sought-after therapeutic target [114]. The structures of pyrazole-based JAKs inhibitors used in cancer treatment are presented in Figure 6.

### 9.1. Ruxolitinib

Ruxolitinib, formerly known as INCB018424, is a selective JAK1 and JAK2 inhibitor with IC_50_ values close to 3 nM. The effect on JAK3 is significantly lower, with an IC_50_ value close to 430 nM [115].

Structurally, ruxolitinib contains a pyrazole ring directly linked to a pyrrolo[2,3-d]pyrimidine scaffold. Based on docking studies, ruxolitinib is considered a type I inhibitor of JAK1 that binds to the DFG_in_ state of the kinase. This interaction is facilitated by the shape complementarity between ruxolitinib and the binding pocket in JAK1. Within the catalytic domain, the pyrrolopyrimidine scaffold aligns with the hinge region and establishes two hydrogen bond connections with Glu957 and Leu959, while the cyclopentane ring is oriented towards the N-lobe and the nitrile group interacts with Lys908 [115]. The crystal analysis of the JAK2 kinase domain grown with ruxolitinib showed a similar binding mode to that described for JAK1, the main interactions being the hydrogen bonds with Glu930 and Leu932 of the hinge region [116].

Ruxolitinib received FDA approval in 2011 for the treatment of myelofibrosis and three years later for the treatment of patients with polycythemia vera. Furthermore, in 2019 and 2021, ruxolitinib obtained FDA approval for the treatment of acute and chronic graft-versus-host disease, a non-neoplastic usage [113]. Since 2022, it is approved as a topical treatment for atopic dermatitis [117].

Baricitinib is also a FDA approved pyrazole derivative structurally similar with ruxolitinib that inhibits both JAK1 and JAK2, but the clinical studies have been focused on the treatment of inflammatory diseases and not cancer [118]. The role of the pyrazole ring in the structure of JAK1 inhibitors can be demonstrated by its use in the structure of povorcitinib (synonym: INCB54707), a PKI in clinical trials for inflammatory diseases [119]. A similar example is izencitinib (TD-1473), a non-selective JAK inhibitor developed for the treatment of inflammatory bowel diseases [120]. Brepocitinib (PF-06700841), also an aminopyrazole derivative, is a JAK1/Tyk2 inhibitor designed as treatment for severe autoimmune diseases [121]. Ropsacitinib (PF-06826647) contains two pyrazole rings directly attached to a pyrazolopyrazine scaffold and can be considered a baricitinib derivative. It is a selective inhibitor of Tyk2, and, currently, there are clinical trials for it as treatment for various autoimmune disorders [30].

### 9.2. Itacitinib

Itacitinib is a JAK1 and JAK2 inhibitor with an IC_50_ value close to 3 nM for JAK1 and around 21 times higher for JAK2. Chemically, it is a derivative of baricitinib, both sharing the 3-(4-(7H-pyrrolo(2,3-d)pyrimidin-4-yl)-1H-pyrazol-1-yl)azetidin-3-yl)acetonitrile scaffold. In a phase III study, itacitinib reduced the total syndrome score in patients with myelofibrosis in a comparable manner with ruxolitinib, but with significantly less incidence of thrombocytopenia [122].

### 9.3. Golidocitinib

Thrombocytopenia and anemia were associated with ruxolitinib treatment as a consequence of the JAK2 inhibition, motivating research into the development of JAK1 selective inhibitors [123]. Golidocitinib (AZD4205) is a highly potent, orally administered inhibitor that specifically targets JAK1. This compound has shown remarkable efficacy in inhibiting tumor growth both in laboratory settings, specifically in T lymphoma cells cultured in vitro, and in animal models with tumor xenografts [117].

The compound was developed based on a 2-amino-pyrimidine derivative substituted with a pyrazole and an indole ring. The introduction of a methyl-piperazine fragment linked to the indole moiety improved JAK1 potency. An ortho substitution on the pyrazole ring (position 3) proved to be important for its selectivity over JAK2 [123].

### 9.4. Gandotinib

Gandotinib is also known as LY2784544 and is an orally bioavailable, relatively selective JAK2 inhibitor. It is a type I inhibitor binding to the ATP pocket of the active conformation of the kinase [124].

The screening of a large collection of compounds lead to the identification of an imidazopyridine derivative with a 3-aminopyrazole scaffold as a potent JAK2 inhibitor. The drug design efforts were concentrated to achieve a better selectivity toward JAK2 compared to JAK3. The core scaffold was kept and appropriate substitution of the imidazopyridazine ring lead to gandotinib [125].

Gandotinib specifically targets the hyperactive JAK2V617F mutant enzyme, a protein found in close to 90% of the polycythemia vera cases and over 50% of the patients suffering from essential thrombocytopenia and myelofibrosis. The preliminary clinical trials (Table S1) demonstrated an acceptable safety and tolerability profile [126].

### 9.5. Ilginatinib

Ilginatinib, also referred to as NS-018, is an orally bioavailable potent inhibitor of JAK2 and Src-family kinases. It has an IC_50_ of 0.72 nM on JAK2, and selectivity ratios of 46-fold, 54-fold, and 31-fold toward JAK1 (IC_50_ = 33 nM), JAK3 (IC_50_ = 39 nM), and Tyk2 (IC_50_ = 22 nM), respectively [127]. It shows higher selectivity for JAK2V617F compared to the wild form.

The analysis of the X-ray co-crystal structure of ilginatinib bound to JAK2 revealed that it established hydrogen bonds with the backbone amino and carbonyl groups of Leu932, which are located in the hinge region, and interacted with the carbonyl group of Gly993 through two distinct hydrogen-bonding interactions. A hydrogen bond was observed between a pyrazine nitrogen atom and a water molecule. Moreover, this water molecule formed a second hydrogen bond with the carbonyl group of Gly993 [128].

## 10. Bcr-Abl Inhibitors

Abl, a non-receptor tyrosine kinase encoded by the Abelson leukemia virus (ABL) oncogene (from the long arm of chromosome 9), is widely expressed in nearly all human tissues inside the nucleus (c-Abl) or cytoplasm of cells, regulating several cytoskeleton functions. The breakpoint cluster region (BCR) gene (found on chromosome 22) encodes the Bcr protein, a tyrosine kinase with cellular signaling function [129,130]. Accidental fusion of BCR to ABL takes place commonly at three points (p185, p210 and p230), giving rise to the chimeric BCR-ABL gene that produces Bcr-Abl proteins (e.g., the P210 protein, a constitutively activated PK).

The fusion protein increases the kinase activity of the original Abl, disrupts signaling pathways, and implicitly promotes abnormal proliferation, with decreased apoptosis, resulting in a clinical outcome that most often translates to various forms of leukemia [129,131]. Its structure distinguishes multiple domains, of which the SH1 region is best conserved, and also harbors the catalytic site from which the dysregulated signaling pathway takes its start; both the biochemistry of the Abl protein as well as the kinase structure and mechanism have been described in great detail [132,133]. Briefly, the kinase consists of the classical N-terminal and C-terminal lobes, connected by a hinge region, while ATP binds through two hydrogen bonds inside a fissure between these two (while specific residues, such as Thr315, play crucial roles in selectivity and resistance) [134].

**Figure 7:**
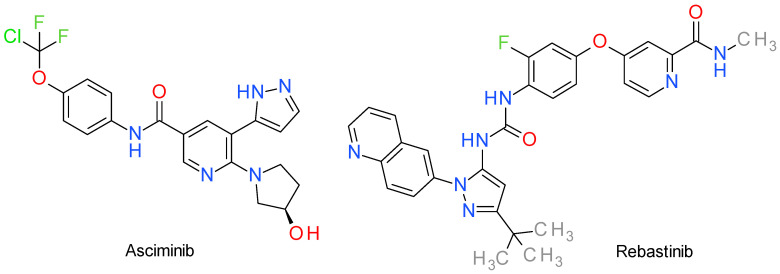
The structures of Bcr-Abl inhibitors Asciminib and Rebastinib.

Even though there are several approved Bcr-Abl inhibitors clinically used, the risk of resistance remains a critical concern, rendering the research in this area highly important [135]. Two Bcr-Abl inhibitors, asciminib and rebastinib, are pyrazole derivatives and their structures are presented in Figure 7.

### 10.1. Asciminib

Asciminib (also presented in literature as ABL001) is an EMA and FDA approved potent, non-ATP competitive, selective allosteric Abl1 inhibitor. It was the first inhibitor in its class to specifically target the myristate (or mysristoyl) pocket of Abl, characterized by a K_d_ of 0.5–0.8 nM and an IC_50_ of 0.5 nM [136] (up to 20 nM by other authors [137]), with the added advantage of activity against clinically meaningful mutations, demonstrating an approximately 65% tumor growth suppression in chronic myeloid leukemia models [138]. Its off-target effects have been appreciated as minimal (no notable GPCR, transporter, ion channel or nuclear receptor interactions) [137], hinting at structural characteristics that may aid selectivity.

In designing asciminib, the cardiovascular adverse effects attributed to hERG activity were alleviated by the use of the pyrazole ring as opposed to pyrimidine [139] or other nitrogen heterocycles (e.g., imidazole in nilotinib) [140]. The amide moiety packed between two aromatic cycles serves as a backbone to facilitate hydrogen bonds with Glu286 and Asp381 of the kinase, eliciting inhibitory activity [140], while the pyrazole ring forms a similar bond with Glu481 and a hydrophobic interaction with Thr453 [136]. The trihalogenated methoxy group has been shown to occupy the myristate pocket of the protein, with one of the fluorine atoms interacting with Leu359, and promote helix-I bending [140,141]. The addition of the hydroxyl group to the pyrrolidine heterocycle increases overall water solubility, although the pyrazole ring also facilitates solubility and oral absorption [140]. Some authors have pointed out that asciminib is a P-gp and BCRP substrate, with a very limited amount being able to successfully cross the blood-brain barrier in murine models [142].

### 10.2. Rebastinib

Rebastinib (identified also as DCC-2036) is an investigational conformational control Bcr-Abl inhibitor for Abl1 (wild-type and T315I mutant, with an IC_50_ of 0.8 nM and 4 nM), as well as other families of proteins (e.g., Src, Lyn, Fgr, Hck, Kdr, FLT3, Tie-2, Axl) [143], which was designed to surpass resistance to the first and second generation of inhibitors in terms of Abl gatekeeper mutations [132]. It exhibits significant anti-proliferative effects on Ba/F3 cells that express either native or mutant forms of Bcr-Abl1, with IC_50_ values ranging from 2 nM to 150 nM and is currently studied for various types of locally advanced or metastatic neoplasms [144].

Structurally, rebastinib follows the scaffold of earlier-developed PKIs in its class, comprised of a head and a tail joined with a linker. Specifically, the linker takes the form of an urea group that facilitates hydrogen bonding with amino acid residues from the C-helix, such as Glu282 and Arg386; the rest of the configuration forms Van der Waals interactions with the hydrophobic clusters of the kinase, leading to the DFG_out_ arrangement, which is further stabilized through the t-butyl attached to the pyrazole ring [132,145,146]. Conclusively, there is evidence in the literature that suggests both DFG_out_ and C-helix-out stabilization is achieved with ligands similar in structure to rebastinib, a conformation which suppresses phosphotransferase activity from the catalytic domain [147,148].

It is currently used in Phase I clinical trials alone, for chronic myeloid leukemia, and in combination with other chemotherapeutics, for patients with locally advanced or metastatic solid tumors [149].

## 11. c-Met Inhibitors

MET (which stands for “mesenchymal-epithelial transition” or as an abbreviation for the mutagen N-methylnitrosoguanidine [150]) is a proto-oncogene and fusion gene discovered in the early 1980s [151] and found at locus 7q31 on human chromosome 7. It encodes the 170–180 kD, two-chain HGFR (hepatocyte growth factor receptor, or simply c-Met), a protein with transmembrane tyrosine-kinase function [152], and it is continuously being researched for its involvement in oncologic pathologies, predominantly of the lung and liver but also breast, ovarian, and GI cancers [153].

**Figure 8:**
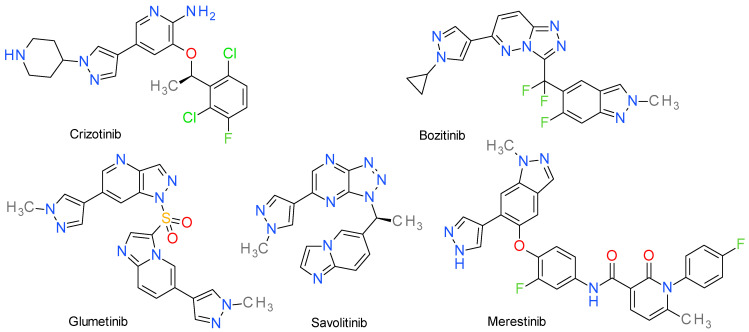
The structures of c-Met inhibitors Crizotinib, Bozitinib, Glumetinib, Savolitinib and Merestinib.

As a therapeutic strategy, targeting c-Met is customarily accomplished with three categories of drugs: multikinase inhibitors, selective c-Met inhibitors and monoclonal antibodies that target either the natural ligands or the receptor itself. Small molecule kinase inhibitors are promising agents due to the relative ease of in silico assisted drug-design and industrial synthesis [154]. The structures of pyrazole-based C-Met inhibitors are presented in Figure 8.

### 11.1. Crizotinib

Crizotinib, also identified in literature as PF-02341066, is an orally-bioavailable, FDA and EMA-approved multikinase inhibitor, described as eliciting an effect at a low nanomolar range on c-Met, ALK (anaplastic lymphoma receptor tyrosine kinase), proto-oncogene tyrosine-protein kinase ROS [155] and VEGFR [156]. ALK is a related receptor tyrosine kinase, belonging to the insulin receptor superfamily of PKs [157].

Pharmacodynamically, the compound is perceived as a type I inhibitor of ALK and I½ B inhibitor of c-Met (although some authors regard it as a type Ia [158]), with the general accepted mechanism being a U-shaped competitive binding in the ATP-specific spot of the kinases. It mirrors ATP interactions with the c-Met protein, e.g., hydrogen bonds with proline, tyrosine, and methionine residues of the hinge region [159]. The selectivity of the compounds is explained by an interaction called π-stacking, between crizotinib’s delocalized π-electron clouds of its aromatic moiety and Tyr1230, a tyrosine residue found inside the activation loop of the kinase [160].

The 2-aminopyridine fraction is pivotal in crizotinib’s activity on c-Met, as the amino group mediates a hydrogen bond with the carbonyl group of Pro1158, while the nitrogen atom inside the pyridine ring binds to the amino group of Met1160. The halogenated 3-benzyloxy part of the molecule confers its potency by ensuring both stability and hydrophobic interactions [158]. The pyrazole fragment functions as a linker that provides an extended conformation and vector for the polar N-substituent (piperidine). The pyrazole is also bound through the narrow lipophilic tunnel surrounded by Ile1084 and Tyr1159 [161]. Crizotinib binds to ALK at Met1199 and Glu1197 through the aminopyridine fragment [101].

Currently limited to its indication in non-small cell lung cancer (NSCLC) that is either ALK positive or ROS positive, crizotinib is actively being researched for its responses in many other types of cancer, such as glioblastoma (although it has a low permeability for the blood-brain barrier, being a P-glycoprotein substrate), gastric and esophageal carcinoma [155], or other GI and haematological malignancies, with regards to its antiangiogenic characteristics [162,163].

### 11.2. Bozitinib

Bozitinib, also identified in literature as PLB-1001, CBT-101, APL-101, CBI-3103, boruitinib or vebreltinib, is a highly selective, ATP-competitive (type I) c-Met inhibitor with a cited IC_50_ value of 8 nM [152]. It provides the advantage of being both orally bioavailable and possessing blood-brain barrier permeability [164]. When preclinically compared to the already approved crizotinib and capmatinib, it proved its superior efficacy in the case of lung cancer models while also strongly inhibiting tumor growth of gastric, lung, hepatic, and pancreatic cell lines [165]. Presently, it is being clinically researched predominantly as monotherapy for various types of cancers (e.g., NSCLC, glioblastoma or solid c-Met expressing tumors).

Although it structurally shares the same pyrazole ring with the approved multikinase inhibitor crizotinib, bozitinib extends one of its pyrazole heterocycle into an indazole scaffold (which has been reported in literature to mediate hydrogen bonds with Tyr1230 and Arg1086 of c-Met and effect a competitive, inhibitory action upon the receptor). In bozitinib, the piperidine of crizotinib is replaced with a cyclopropyl group, attached to its other isolated pyrazole ring. Additionally, the N-aminopyridine fragment of crizotinib is replaced by the triazolo[4,5-b]pyrazine moiety, described at the beginning of the last decade as possessing “exquisitely selective” ATP-competitive c-Met kinase inhibition properties [154,166].

### 11.3. Glumetinib

Glumetinib is also referred as SCC244 or gumarontinib and it is a type II ATP-competitive c-Met inhibitor with oral bioavailability and high selectivity in the range of tenths of a nanomolar (IC_50_ value of 0.42 ± 0.02 nM) [167]. It is currently investigated for indications regarding lung and solid cancers [168,169].

The available literature [170,171] on the drug is currently lacking any in-depth structure activity analyses. One article suggests that the N-methylpyrazol-4-yl group might offer a greater coplanarity with the core moiety, as seen in other c-Met inhibitors. The imidazopyridine and pyrazolopyridine bridged by the sulfonyl group increase the π-π stacking with Tyr1230, while lowering the overall lipophilicity of the molecule, thus enhancing the metabolic stability in both microsomal environment and the hepatocyte [172].

### 11.4. Merestinib

Merestinib (identifier: LY2801653) is presented as a type II ATP-competitive multikinase inhibitor (although some authors class its mechanism of action as ATP non-competitive) [150,173,174]. Historically, the molecule was structurally designed as a c-Met inhibitor, but was later screened over a broader panel of kinase targets. Its array of targets include not only c-Met, but also RON, AXL, FLT3, c-Kit, MERTK (MER proto-oncogene tyrosine kinase), Tie-2 (TEK), ROS1, NTRK (neurotrophic tropomyosin receptor kinase) 1/2/3, DDR (discoidin domain receptor) 1/2, and MKNK (MAP kinase interacting serine/threonine kinase) ½ [154,170,175]. With nanomolar ranges of inhibitory concentrations for most of the previously-enumerated kinases, it is seen as a significant and potent inhibitor of c-Met (IC_50_ value of 4.7 nM) [152].

Merestinib encompasses both a pyrazole and an indazole heterocycle and there is little available recent literature that reviews its structure-analysis relationship. The molecule has been reported to be active against certain c-Met mutations that drive resistance against tepotinib or capmatinib. The distance between merestinib’s pyrazole and the c-Met’s Val1228 was shown to be 4 Å smaller than is the case for capmatinib’s quinoline heterocycle, hinting at a stronger interaction in this region. Apart from the canonical 4-fluorophenylamine which ensures penetration within the receptor’s hydrophobic pocket, the co-crystal structure of c-Met and merestinib reinforces its base mechanism of action, which is that of stabilizing the protein in its inactive DFG_out_ state, and further analyses revealed that the molecule has an increased occupancy time (t_½_ of ~8.5 h) while also presenting the advantage of a greater mutation tolerance, which is an advantage over type I inhibitors [176,177,178].

It is under investigation for its antineoplastic properties in advanced or metastatic cancers, especially of biliary, colorectal, lung, or pancreatic origins, as well as solid tumors and leukemias––pathologies where the inhibition of any of the abovementioned plethora of kinases exerts a crucial and beneficial effect, either clinically or pre-clinically [154,174,179,180,181].

### 11.5. Savolitinib

Savolitinib, found in the available literature under several aliases (Volitinib, AZD6094, HMPL-504, HM-5016504) is a novel and still experimental candidate that presents a tyrosine kinase inhibitory profile and entered the clinical landscape (Table S1) at the beginning of the last decade for patients with advanced solid tumors [182]. After screening more than 265 kinase targets, it has been described as a potent and selective (type I) inhibitor of the phosphorylated form of the MET receptor, with an IC_50_ value of 4 nM [183]. Trials that are ongoing or have ended explored its involvement in the treatment of colorectal, renal, gastric, lung, and central nervous system (CNS) tumors [153,184,185,186].

Savolitinib is often regarded as different from other common second generation c-Met inhibitors by lacking the quinoline moiety that has been canonically reported as conferring selectivity (and also toxicity through aldehyde oxidase metabolism). It is built upon the 1,2,3-triazolo-4,5-b-pyrazine scaffold, with the pyrazole ring being situated contralaterally to the hydrophobic pole [187]. It has been hypothesized that savolitinib interacts similarly to the other c-Met inhibitors by adopting the predicable U-shape around kinase’s Met1211 while providing the necessary hydrogen bonds or π-stacking with amino acids also found in the activation loop. The molecule has been strategically fashioned to contain the small methyl group that blocks the methylene linker from being metabolically oxidized. The role of the pyrazole moiety has been reported in one recent paper as having a positive influence on lipophilicity, ensuring a decreased clearance, while safeguarding potency [187,188].

## 12. EGFR Inhibitors

The epidermal growth factor receptor (EGFR) is a transmembrane glycoprotein that plays a crucial role in cell signaling, promoting cell survival, growth, and division. EGFR consists of several domains, including an extracellular ligand-binding domain and a cytoplasmic tyrosine kinase domain [189]. Pharmacologically, EGFR is targeted by monoclonal antibodies that bind to the extracellular domain and block the kinase activation produced by the various growth factors or by small molecules capable of entering the cell and interacting with the intracellular tyrosine kinase domain of EGFR [190]. Based on their mechanism, there are two major groups of EGFR inhibitors. The first type consists of inhibitors that reversibly bind to the EGFR’s kinase domain and compete with ATP for binding, such as erlotinib and gefitinib, while the second type entails inhibitors form a covalent bond with the cysteine residue leading to an irreversible inhibition [191]. Many cancers harbor a T790M mutation at the highly conserved gatekeeper, and the covalent inhibitors were designed to circumvent this problem [192].

### 12.1. Lazertinib

Lazertinib, also referred as YH25448, is a brain-penetrant, irreversible EGFR inhibitor that targets both the EGFR T790M mutation and the activating mutations Del19 and L858R while having less effect on wild type-EGFR, and, therefore, having diminished side effects [193].

**Figure 9:**
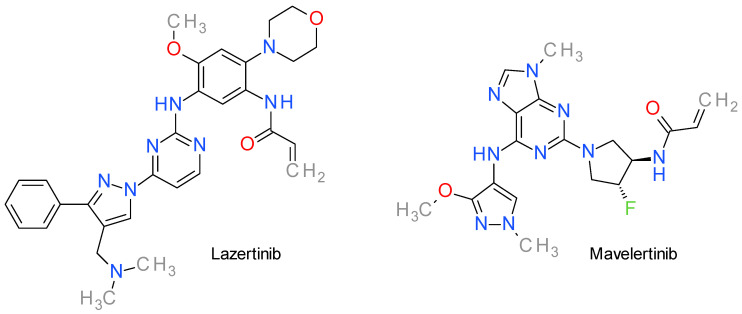
The structures of EGFR inhibitors Lazertinib and Mavelertinib.

The compound targets the Cys797 residue in the ATP-binding site of the EGFR kinase domain through its acrylamide warhead and irreversibly inhibits the kinase activity. The 2-aminopyrimidine moiety (Figure 9) binds to the hinge residue Met793 through hydrogen bonding, while the phenyl substituent in the pyrazole ring points to the gatekeeper residue Met790 and the morpholine ring faces the solvent exposure region. The 2-aminopyrimidine scaffold is considered the pharmacophore and confers selectivity for the mutated forms of EGFR in contrast to the wild type [194]. The pyrazole ring substitutes the corresponding indole ring of osimertinib and acts as a linker for the phenyl and for the dimethylaminomethyl fragments [195].

Lazertinib is a third generation EGFR inhibitor approved in some countries, such as the Republic of Korea, for the treatment of locally advanced or metastatic NSCLC in patients with T790M mutation [196].

### 12.2. Mavelertinib

Mavelertinib, also known as PF06747775, is a third-generation covalent inhibitor of EGFR, with low brain permeability and promising results in phase I clinical trials for the treatment of EGFR-driven NSCLC [195,197]. It exhibits potent EGFR activity against common mutants such as exon 19 deletion (Del), L858R, and double mutants T790M/L858R and T790M/Del, while sparring wild-type EGFR [198]. As with other EGFR inhibitors, it binds covalently to the Cys797 residue in the ATP-binding site, and is therefore less effective on the emergence of the C797S mutation [199].

## 13. PDGFR Inhibitors

The platelet-derived growth factor receptor (PDGFR) belongs to the type III family of receptor tyrosine kinases together with c-KIT, c-FMS, and FLT3. They are related to a lesser degree to the VEGFR [200]. Elevated levels of PDGFRs have been consistently associated with several types of cancer, such as glioma, Kaposi’s sarcoma, prostate cancer, or GI stromal tumors [200].

### Avapritinib

**Figure 10:**
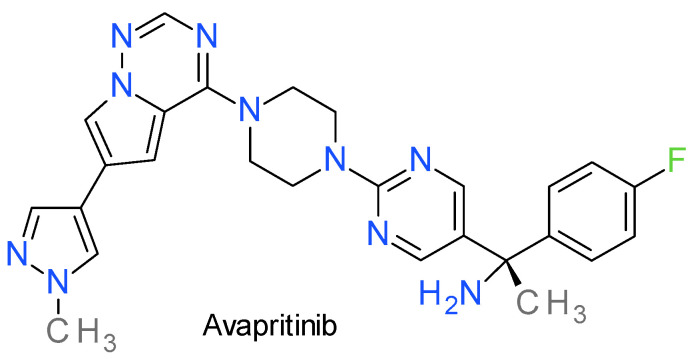
The structure of Avapritinib.

Avapritinib, also reported as BLU-285, is an orally bioavailable PKI against mutant forms of PDGFRA and c-KIT. Chemically, the pyrazole ring is directly bonded to a pyrrolotriazine scaffold (Figure 10). It was designed as a ATP-competitive inhibitor, targeting preferentially the active kinases conformation [201]. In vitro, avapritinib has demonstrated potent activity on activation loop mutants, such as KIT-D816V and PDGFRA-D842V, on the juxtamembrane domain mutations, such as KIT exon 11, and on the ATP binding pocket mutations, such as KIT exon 13 and 14 [202]. The D842V mutation has been primarily observed in patients with systemic mastocytosis and GI stromal tumors (GIST), cancers that are resistant to imatinib or sunitinib [203].

Avapritinib is approved by FDA and EMA as monotherapy for the treatment of adult patients with inoperable or metastatic GIST harboring the PDGFRA exon 18 mutation, including PDGFRA D842V mutation, and for the treatment of adult patients with aggressive systemic mastocytosis, systemic mastocytosis with an associated haematological neoplasm, or mast cell leukaemia after at least one systemic therapy [203].

## 14. FGFR Inhibitors

The fibroblast growth factor receptor (FGFR) family, comprising FGFR1 to FGFR4, encompasses four members that exhibit high structural similarity. As with other receptor tyrosine kinases, FGFRs are located on the cell membrane and can be triggered by external signals, with the fibroblast growth factors (FGFs) serving as the natural ligands [204,205]. Several FGFR inhibitors share a pyrazole ring, with the most important being AZD4547, an oral inhibitor selective for FGFR1, 2, and 3 [206]; Ly2874455, which is a pan-FGFR inhibitor [207]; zoligratinib (synonym: Debio 1347) [208]; and the commercially available erdafitinib [209].

### Erdafitinib

Erdafitinib, also referred as JNJ-42756493, is a pan-FGFR inhibitor. Following enzyme binding, it inhibits FGFR phosphorylation and suppression of FGFR-related signal transduction pathways, thus inhibiting tumor cell proliferation and cell death in FGFR-overexpressing tumor cells [210].

**Figure 11:**
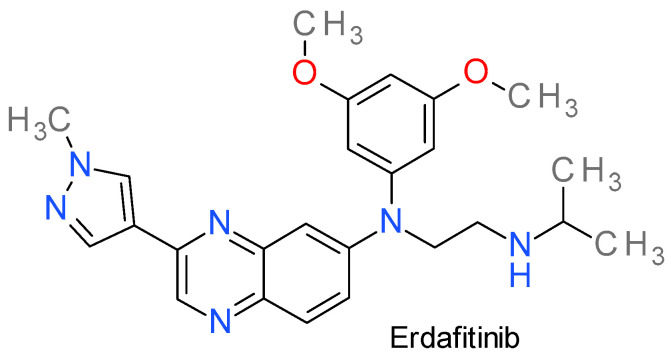
The structure of Erdafitinib.

It contains a 3-(1-methyl-1H-pyrazol-4-yl)-quinoxaline scaffold (Figure 11) and is a reversible, type I½ inhibitor that bind FGFRs in the inactive DFG_in_ conformation A SE VERIFICA DACA NU E DFG-OUT! in an ATP-competitive manner [211]. Molecular docking analysis showed that erdafitinib displays hydrophobic interactions with Leu478, Val486, Lys508, Val555, Leu624, and Asp635 residues of FGFR3, and forms hydrogen bonds with Ala558 and Asn562 residues, respectively [211].

The K_d_ values towards the four PK members of the FGFR family fall in the 0.24–2.2 nM range, and the IC_50_ values between 1.2–5.7 nM, respectively. Erdafitinib exhibits a lower affinity against VEGFR2 kinase, with K_d_ and IC_50_ values of 36.8 nM and 6.6 nM, respectively. This is the reason why it presents fewer adverse reactions due to VEGFR2 inhibition (such as diarrhea, vomiting, and fatigue [212]). The evidence of its antitumor activity was demonstrated in several FGFR-expressing cell lines and in animal models with FGFR translocations or amplifications [212,213].

Erdafitinib was found to successfully reverse multidrug resistance (MDR) caused by ABCB1, also known as P-glycoprotein 1. Interestingly, erdafitinib did not have an impact on ABCG2-mediated MDR. The expression and cellular localization of ABCB1 remained unaffected by the drug [214].

It has been approved by the FDA since 2019 for adult patients with locally advanced or metastatic bladder cancer with susceptible FGFR3 or FGFR2 genetic alterations that has progressed during or following prior platinum-containing chemotherapy [210]. It is currently under investigation for hepatocellular carcinoma, breast cancer, NSCLC, and prostate cancer [214].

## 15. RET Inhibitors

RET, which stands for “rearranged during transfection”, is a receptor tyrosine kinase responsible for binding neurotrophic factors. In certain types of human cancers, genetic changes occur in the RET gene, resulting in activating point mutations or rearrangements that give rise to chimeric oncoproteins where the kinase domain becomes fused with the N-terminal region of heterologous proteins. The first generation of RET inhibitors are multikinase inhibitors, with important off-target toxicities prompting the development of potent inhibitors with high selectivity against RET [215].

### Pralsetinib

Pralsetinib (BLU-667) is a highly potent RET inhibitor specifically developed to target and inhibit resistance mutations in RET as well as the wild-type enzyme, demonstrating subnanomolar potency with IC_50_ values of 0.3 nM and 0.4 nM for RET-V804L and RET-V804M, respectively [216]. It inhibits at higher IC_50_ values other tyrosine kinase, such as VEGFR2, FGFR2, and JAK2 [217].

**Figure 12:**
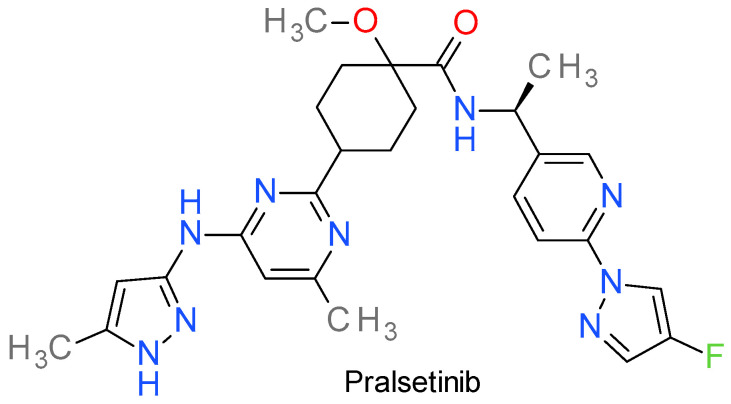
The structure of Pralsetinib.

It is an aminopyrazolyl substituted pyrimidine (Figure 12) that functions as a type I inhibitor by binding the active site through two hydrogen bonds between the pyrazole ring and the Ala807 and Glu805 residues. Type I PKIs usually pass through the gate and bind both the front and back clefts, or only to the front cleft (type IB). Conversely, pralsetinib attaches to the front cleft without passing through the gate, while wrapping around the region outside the gate wall formed by the side chain of Lys758 and targeting the other end in the pocket located in the back cleft [218]. The mutations L730V/I are resistant to pralsetinib, with almost 60 fold higher IC_50_ values, because they produce a steric clash with the cyclohexane fragment at the roof of the solvent-front region [219].

In September 2020, the FDA granted accelerated approval for pralsetinib to treat adult patients with metastatic fusion RET positive NSCLC, and on December 2020, for advanced or metastatic medullary thyroid cancer with genetically defective RET [217]. Pralsetinib exemplifies the concept of personalized medicine in cancer treatment, with the drug being selected based on the specific genetic characteristics of the patient’s tumor.

## 16. Inhibitors of Various Other Tyrosine Kinases

### 16.1. Mivavotinib

Mivavotinib, also known as TAK-659, is a highly potent, selective, reversible, and orally available dual inhibitor of FLT3 and of the spleen tyrosine kinase (Syk), and it is in clinical development for the treatment of patients with advanced solid tumors or hematologic malignancies. Syk is a cytosolic non-receptor tyrosine kinase predominantly found in hematopoietic cells, and it is a crucial component in the signaling pathway of the B-cell receptor [220].

**Figure 13:**
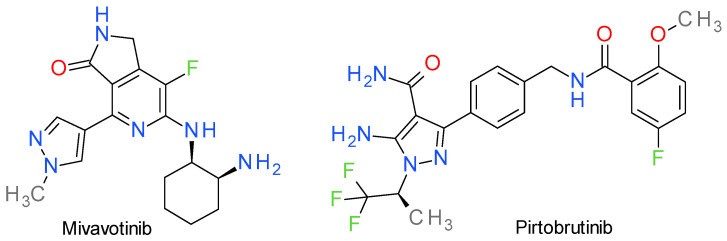
The structure of Mivavotinib, a dual inhibitor of FLT3 and Syk, and the structure of Pirtobrutinib, a clinically approved BTK inhibitor.

Mivavotinib binds to the hinge region of Syk in its DFG_in_ conformation through the lactam core, which makes two direct hydrogen bonds with the hinge through Glu449 and Ala451. The amines of the cyclohexyl ring also interact with Asp512, Asn499, and Arg498, while the methyl pyrazole ring (Figure 13) occupies the lipophilic region, with the nitrogen forming a hydrogen bond with a water molecule. The compound was developed starting from a 1,2-dihydro-3H-pyrrolo[3,4-c]pyridin-3-one derivative 4-substituited with an aniline fragment. The pyrazole ring was used to replace the lead’s aniline in order to reduce the lipophilic character, the CYP liability, and the interaction with hERG [220].

Despite the good results on hematopoietic-derived cell lines and in murine models [221], a phase I clinical study in patients with relapsed/refractory acute myeloid leukemia indicated modest results [221].

### 16.2. Pirtobrutinib

Pirtobrutinib, also known as LOXO-305, is a highly selective, orally bioavailable, reversible, Bruton’s tyrosine kinase (BTK) inhibitor of both wild type and C481S mutant, with similar IC_50_ values in both enzymatic and cell-based assays [222]. It is the leading member of a new generation of BTK inhibitors that non-covalently inhibit the kinase activity without a direct interaction with Cys481, unlike the covalent inhibitors that target this residue. This mechanism renders pirtobrutinib active in ibrutinib-resistant chronic lymphocytic leukemia (CLL), and this is the reason why Cys481S mutant variants of this tyrosine kinase are resistant to treatment with previous generations of BKT inhibitors [223].

Pirtobrutinib demonstrated more than 100-fold selectivity against almost all of the 350 kinases tested in vitro. This high selectivity gives it a superior safety profile when compared to covalent BTK inhibitors [224]. Chemically, it is based on the aminopyrazole carboxamide scaffold (Figure 13), a structure designed to replace the 4-aminopyrazolopyrimidine structure of ibrutinib [225]. It was probably inspired by the structure of zanubrutinib, by opening the tetrahydropyrazolo(1,5-a)pyrimidine ring. Pirtobrutinib fixes itself in the ATP-binding site, forming three hydrogen bond interactions with the backbone of Glu475 and Met477 in the hinge region. It also forms water-mediated hydrogen bonds with Lys430 and Asp539, and an edge-to-face π-stacking interaction with Phe540. In multiple cell assays, pirtobrutinib prevented Tyr551 phosphorylation in the kinase activation loop, probably because it stabilizes BTK in a closed, inactive conformation [222].

It is approved by FDA in the Accelerated Approval Program since January 2023 and by EMA in a conditional marketing authorization in April 2023 for the treatment of adult patients with relapsed or refractory mantle cell lymphoma after at least two lines of systemic therapy, including another BTK inhibitor [226].

## 17. Conclusions

The review of the structures of PKIs clinically tested revealed 42 compounds that contain an unfused pyrazole ring. For comparison, a similar search based on the pyrazole isomer, the imidazole ring, revealed 10 compounds. Searches on the related pyrrole ring prompted only 7 compounds, highlighting the importance of the pyrazole scaffold for this class of compounds. There are also important PKIs that contain pyrazole-fused scaffolds such as indazole, pyrazolo[4,3-b]pyridine, or pyrazolo[1,5-a]pyrimidine, but because of the different electron distribution, aromatic profile, hydrogen bonding capacities, and geometric particularities of each scaffold, we decided to focus only on the unfused pyrazoles in order to have a more accurate picture of its role in the design of PKIs.

The pyrazole scaffold offers several advantages for the design of PKIs. Its aromatic nature and ability to serve as hydrogen bond acceptor or donor facilitates interactions with key residues in the kinase’s active site or binding pockets. The pyrazole ring can participate in π-stacking interactions with aromatic residues in the kinase active site, enhancing binding affinity.

The compounds reviewed here inhibit a large diversity of PKs, not being restricted to a single family of kinases. The majority of the compounds have a relative selectivity toward one single PK or limited to close related kinases, indicating the usefulness of the pyrazole ring in the development of PKIs. The structure and mechanism of action analyses revealed that the pyrazole ring can function as an analogue of the adenine fragment in ATP and bind competitively to its site, or that it can be used as a linker to provide the proper conformation for the inhibitor. It would be also wrong to assume that only the pyrazole ring is important, since we can easily observe that each compound reviewed here contains at least another cyclic structure.

In summary, the pyrazole ring plays a crucial role in the development of anticancer therapies targeting specific PKs. It can serve as an ATP analogue, competitively binding to the ATP binding site and as a linker, facilitating the proper conformation for effective inhibition. However, it is important to consider the overall structure and composition of the inhibitors as additional structural elements may uniquely contribute to their activity and selectivity.

## Data Availability

Not applicable.

## Supplementary Materials

The following supporting information can be downloaded at: https://www.mdpi.com/article/10.3390/molecules28145359/s1, Table S1: Clinical trials and approved status for the pyrazole-based protein kinase inhibitors.
