# Supplementary material for: The Importance of the Pyrazole Scaffold in the Design of Protein Kinases Inhibitors as Targeted Anticancer Therapies
Source: Molecules. 2023 Jul 12;28(14):5359. doi: 10.3390/molecules28145359 (PMC10385367; doi:10.3390/molecules28145359)
Supplement: Supplementary file 1 [file molecules-28-05359-s001.zip › molecules-2484784-supplementary.pdf]

molecules

Review

The Importance of the Pyrazole Scaffold in the Design of Protein Kinases Inhibitors as Targeted Anticancer Therapies

George Mihai Nitulescu [1], Gheorghe Stancov [1], Oana Cristina Seremet [1], Georgiana Nitulescu [1]*, Dragos Paul Mihai [1], Cosmina Gabriela Bratu Duta [1], Stefania Felicia Barbuceanu [1], and Octavian Tudorel Olaru [1]

[1] Faculty of Pharmacy, "Carol Davila" University of Medicine and Pharmacy, Traian Vuia 6, Bucharest

Tabel S1. Clinical trials and approved status for the pyrazole-based protein kinase inhibitors.

Protein kinase inhibitor	Clinical trial code as monotherapy	Clinical trial code as combination therapy	Major cancer type	FDA first approval	EMA first approval
Afuresertib	-	-	Hematologic<br>Solid tumours	-	-
Uprosertib	-	NCT01902173	Melanoma<br>Endometrial<br>Solid	-	-
Tozasertib	-	-	Colorectal<br>Advanced Solid Tumors	-	-
Ilorasertib	-	-	Advanced Solid Tumors<br>Hematologic	2011 (orphan status - acute myeloid leukemia)	2011 (orphan status - acute myeloid leukemia)
Barasertib	-	-	Hematologic	-	-
Pexmetinib		NCT04074967	Myelodysplastic Syndromes<br>Renal Cell Carcinoma<br>Melanoma<br>Solid Tumor<br>Non-small Cell Lung Cancer<br>Head and Neck Squamous Cell Carcinoma	-	-

Ravoxertinb			Non-Small Cell Lung Cancer<br>Metastatic Colorectal Cancer<br>Metastatic Non Small Cell Lung Cancer<br>Metastatic Cancers<br>Melanoma<br>Solid Tumor	-	-
Encorafenib	NCT01436656	NCT03843775<br>NCT05270044<br>NCT04390243<br>NCT01909453<br>NCT04655157<br>NCT05195632<br>NCT05217446<br>NCT04673955<br>NCT04061980<br>NCT04074096<br>NCT04324112<br>NCT05026983<br>NCT01543698<br>NCT04017650<br>NCT04657991<br>NCT02834364<br>NCT04045691<br>NCT03839342<br>NCT05576896<br>NCT03693170<br>NCT05004350<br>NCT03235245<br>NCT04526782<br>NCT03898908<br>NCT04221438<br>NCT04511013<br>NCT03915951<br>NCT04720768<br>NCT05203172<br>NCT04607421<br>NCT05308446<br>NCT02675946<br>NCT03864042<br>NCT04741997<br>NCT03973918<br>NCT02910700<br>NCT02631447<br>NCT04800822<br>NCT05039177<br>NCT05538130	GI<br>Melanoma<br>Solid tumours	2018 (BRAF-mutant advanced, unresectable or metastatic melanoma, comb. with binimetinib)	2018 (BRAF-mutant advanced, unresectable or metastatic melanoma, comb. with binimetinib)

		NCT05260684<br>NCT04585815<br>NCT05097599<br>NCT04801966<br>NCT05019534<br>NCT03563729<br>NCT03026517<br>NCT05443087<br>NCT05725200<br>NCT02382549			
Prexasertib			Ovarian cancer<br>Fallopian tube cancer<br>Malignant peritoneal neoplasm	-	-
Voxtalisib			CNS<br>Lung<br>Hematologic<br>Breast<br>Ovarian	-	-
Ruxolitinib	146 Active CTs		Hematologic, varied localizations and indications beyond cancerous pathologies	2011 (intermediate or high-risk MF)	2008 (orphan, myelofibrosis)
Itacitinib	NCT04640025<br>NCT04358185	NCT03697408	Myelofibrosis<br>Hepatic<br>Blood	-	-
Golidocitinib	NCT04105010<br>NCT03450330<br>NCT04225208<br>NCT03728023	NCT05486949	Lymphoma<br>Lung	-	-
Gandotinib	NCT01594723	-	Hematologic	2011 (orphan, myeloproliferative disorders)	
Ilginatinib	NCT04854096	-	Myelofibrosis	-	-
Asciminib	NCT04795427<br>NCT04925479<br>NCT04216563<br>NCT04948333<br>NCT05456191<br>NCT03106779<br>NCT05384587<br>NCT04666259<br>NCT04971226<br>NCT04877522<br>NCT05421091	NCT05143840<br>NCT03595917<br>NCT04492033<br>NCT03578367<br>NCT04838041<br>NCT03874858<br>NCT03906292<br>NCT02081378	Hematologic	2021 (accelerated approval for Philadelphia chromosome-positive chronic myeloid leukemia (Ph+ CML) in chronic phase (CP))	2022 (approved asciminib for the treatment of adult patients with Ph+ CML-CP previously treated with two or more TKIs)
Rebastinib		NCT03717415<br>NCT03601897	Locally Advanced or Metastatic Solid Tumor	-	-

			Chronic Myeloid Leukemia<br>Breast		
Crizotinib	51 active CTs where crizotinib isn't used in combination	NCT01979536<br>NCT03947385<br>NCT04292119	Brain, Blood, Lung, GU, GI, Solid, Agnostic	2011 (accelerated approval for ALK-positive locally advanced or metastatic NSCLC)	2012 (previously treated ALK-positive advanced NSCLC)
Bozitinib	NCT03175224		Lung, Solid, Agnostic	2022 (orphan designation - solid cancers, unspecified)	-
Glumetinib	NCT04270591	NCT04797702	Lung	2022 (orphan designation) (NSCLC) with MET genomic aberration)	-
Merestinib	NCT02920996	NCT02711553<br>NCT02791334	Biliary/hepatic<br>Lung<br>Leuk<br>Pancreatic	2017 (orphan designation Biliary tract cancer)	-
Savolitinib	NCT03598244<br>NCT04923945<br>NCT04923932<br>NCT03091192<br>NCT03592641<br>NCT02897479<br>NCT03385655	NCT03778229<br>NCT05261399<br>NCT04606771<br>NCT05043090<br>NCT05374603<br>NCT05009836<br>NCT05015608<br>NCT05620628<br>NCT02761057<br>NCT02819596<br>NCT03944772<br>NCT02143466<br>NCT03833440<br>NCT02117167	Renal<br>Lung<br>CNS<br>GI<br>Prostate	-	-
Lazertinib	NCT05338619<br>NCT05469022		Lung<br>Solid	-	-
Avapritinib	NCT03580655<br>NCT03731260<br>NCT04773782<br>NCT04825574<br>NCT04254939<br>NCT04771520	NCT04908176	GI<br>Hematologic<br>CNS<br>Solid<br>Breast	2020 (Gastrointestinal stromal tumor)	2020 (Gastrointestinal stromal tumor)
Erdafitinib	NCT02699606<br>NCT02365597<br>NCT04083976<br>NCT05316155<br>NCT04172675<br>NCT03390504<br>NCT03473743	NCT03238196<br>NCT04963153<br>NCT05564416<br>NCT03732703	Solid<br>GU<br>Bone<br>Breast<br>Hematologic	2019 (second-line treatment of bladder cancer with FGFR3 or FGFR2 gene aberrations)	-

Pralsetinib	NCT04754425 NCT03999515 NCT02465060 NCT03155620 NCT04760288 NCT03037385 NCT04222972 NCT04697446 NCT05170204 NCT04302025 NCT05159245 NCT04632992 NCT04589845		Thyroid, Lung, Solid tumours	2020 (metastatic RET fusion-positive NSCLC)	2021 (RET fusion-positive advanced NSCLC not previously treated with a RET inhibitor)
Mivavotinib			Lymphoma	(2019, orphan, Follicular Lymphoma)	(2018, orphan, Acute Myelogenous Leukemia)
Pirtobrutinib	NCT05317936 NCT05023980 NCT05254743 NCT04662255 NCT03740529 NCT04849416 NCT05024045 NCT04666038	NCT04965493 NCT05529069 NCT05536349 NCT05677919	Leukemia Lymphoma Macroglobulinemia Myeloma	2020 (orphan, chronic lymphocytic leukemia)	2021 (orphan, mantle cell lymphoma)

the completed/suspended studies were not included in the table.
